# A Quantitative Comparison of the Counting Significance of van Hove Integral Spectroscopy and Quasielastic Neutron Scattering

**DOI:** 10.1038/s41598-020-63193-3

**Published:** 2020-04-14

**Authors:** Antonio Benedetto, Gordon J. Kearley

**Affiliations:** 1 School of Physics, University College Dublin, Dublin 4, Ireland; 2 School of Chemistry, University College Dublin, Dublin 4, Ireland; 3 Conway Institute of Biomolecular and Biomedical Research, University College Dublin, Dublin 4, Ireland; 4 Department of Sciences, University of Roma Tre, Rome, Italy; 5 Laboratory for Neutron Scattering, Paul Scherrer Institute, Villigen, Switzerland

**Keywords:** Techniques and instrumentation, Chemistry, Physics

## Abstract

We have recently proposed a new method to access system dynamics via neutron scattering based on measuring the elastic scattered intensity: By varying the energy band-width that impinges on the sample (also known as instrumental energy resolution), the purely elastic-scattering from this variation is the running time-integral of the intermediate scattering function (I(t)) [Benedetto and Kearley, Sci. Rep. 9, 11284, 2019]. In this correspondence we denote our method “vHI”, which stands for “van Hove Integral”. The method is now widely accepted as “valid” and here we focus on the efficiency of the vHI method compared with the standard quasi-elastic neutron scattering (QENS) method. We use a numerical Monte-Carlo simulation of an instrument that is equally capable of measuring QENS and vHI under identical conditions. For an “experiment” in which the same number of neutrons enter the instrument, we present comparisons between QENS and vHI at three levels of data-reduction. Firstly, at the raw-data level vHI achieves 100 times more neutrons at the detector than QENS. Secondly, vHI has a factor of 2 less statistical error, which would translate to an overall gain of 4 for vHI in counting-time. Lastly, we compare the distortions caused in obtaining the final I(t) via time-Fourier transform (QENS) and polynomial time-derivative (vHI). Here, the statistical error is 10 times smaller for vHI. This last comparison is the most important result where the 10 times smaller residual for vHI gives a net gain in counting time of 100 better than QENS to obtain the same underlying dynamics of the system under study.

## Introduction

The study of dynamical behaviour at an atomic level plays a major role in understanding the microscopic mechanisms of materials of interest, from simple chemical molecules up to complex biomolecules. Often, structural information alone cannot provide the required level of understanding. A clear example is in biophysics, where the dynamics of enzymes has been shown to be as important as the knowledge of their structure to understand their biochemical function^1–8^.

Among the available techniques, neutron scattering is unique in probing the motion of atomic nuclei, rather than measuring the response of the electrons to the nuclear dynamics, which is the case for most other spectroscopies. This direct type of interaction makes the comparison between neutron scattering and classical molecular dynamics simulations a successful and straightforward combination^9–11^.

A common variant of neutron scattering that is routinely used to access system dynamics is quasi-elastic neutron scattering (QENS)^5,6,11–16^. The output is an energy-spectrum of the number of neutrons scattered by the sample as a function of energy transfer, making QENS *de facto* an “inelastic” method for dynamics. The term ‘quasi-elastic’ derives from the study of diffusive rather than periodic dynamics, resulting in a quasi-elastic broadening of the elastic peak rather than inelastic peaks, respectively. It follows that the information required from a QENS experiment is in the time rather than the frequency domain, so that it is usual to fit the measured QENS signal with model functions that can easily be transformed to the time domain, to get I(t). This transformation can also be made directly with a numerical time-Fourier transformation (time-FT) of the raw data, but truncation and statistical errors can lead to oscillations in the resulting I(t). I(t) is known as “van Hove intermediate scattering function”, and contains all the information of the dynamics of the system^12^ - accessing it directly in the time domain is the dream of many experimentalists.

An alternative approach that has been proposed a number of times is to measure the “elastic” scattering to get some (overall) measure of the system dynamics^17–31^. We recently showed a new method based on elastic scattering, our method being unique in that it accesses I(t) directly^32^. The basic principle is straightforward. We use two energy-band filters, centred at the same energy, with the first, F1, between the neutron source and the sample, and the second, F2, between the sample and the detector. The function obtained by holding the width of F2 at a narrow value, and varying the width of F1 between this narrow value and some higher value, is the running time-integral of I(t). Due this correspondence we denote our method “vHI”, which stands for “van Hove Integral”. The previous elastic-based attempts failed to access I(t) because they were based on existing spectrometers in which F1 and F2 are effectively coupled to optimise the counting efficiency of spectroscopy in the energy domain.

The vHI method is now widely regarded as validated, but the question of relative efficiency of vHI and QENS is still open. We address that question in this letter by comparing their total counts and the statistical errors at the same experimental conditions. To do so we develop a Monte-Carlo (MC) instrument simulation that can run QENS or vHI under as near identical conditions as possible. Being an elastic approach we expect our vHI method to have a considerable gain in count-rate. However, because it is an integral method, not all of the extra counts will translate into statistical relevance. In this letter we show quantitatively that there remains a significant statistical advantage to the vHI method over QENS. Here, we compare our vHI method only with QENS, not with other elastic scattering approaches.

## Aim of this Letter and Methodology

**Figure 1:**
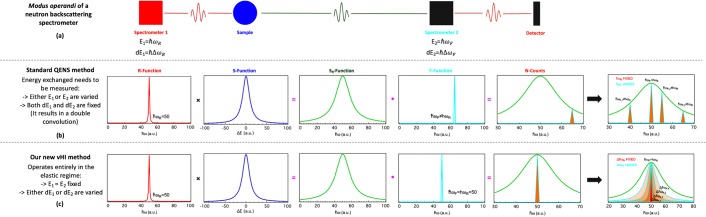
(**a**) The most general layout/*modus operandi* of a neutron backscattering spectrometer in which the interplay between the primary and the secondary spectrometers enables the measurement of either the QENS spectrum (**b**) or the vHI profile (**c**). QENS needs to compute energy exchange and thus operates in the “inelastic” regime with fixed energy resolution. vHI is fundamentally different, it operates in the purely “elastic” regime. For both QENS and vHI there are three constant quantitates and one variable. Here, for QENS dE_1_, dE_2_ and E_2_ are constant and E_1_ is varied. For vHI E_1_, E_2_ and dE_2_ are constant and dE_1_ is varied. We made this choice for simplicity, although other equivalent choices are possible.

Ideally, a comparison of QENS with vHI requires the QENS spectrum and the vHI profile of the same system to be measured with the same instrument. In practice this may never be achievable since the two methods would be optimised in different ways. We take a pedagogic approach here by basing the *in silico* instrument on existing backscattering QENS spectrometers as illustrated in Fig. 1, which could also used for vHI^22,32^. The *in silico* instrument changes between the QENS and vHI modes by simply altering the way in which the primary monochromator is scanned. For QENS the band-width of the primary monochromator (dE_1_) is held constant, whilst the energy of the band-centre (E_1_) is varied to obtain the energy QENS spectrum. For vHI the converse is used. The position of the band-centre on the primary monochromator is held constant, and its energy band-width is varied to obtain the elastic intensity as a function of (the observation) time, effectively the vHI profile. For both methods, the band-width (dE_2_) and the energy of the band-centre (E_2_) of the secondary monochromator are held constant. Clearly, by using the same number of incident neutrons for each mode, we get “experimental data” that can be compared on an equal footing.

### The Monte-Carlo in-silico spectrometer for both QENS and vHI

A neutron beam is simulated with an energy band-width equal to the maxima (energy-loss and energy-gain, max{|∆E |}) of QENS spectrum and centred at the elastic energy. The energy band is divided into n = 2048 channels and neutrons are generated with a Gaussian weighted random distribution. The Gaussian half-width at half-maximum (HWHM) corresponds to the primary resolution at each configuration, dE_1_. This width is held constant at the narrowest value for the QENS-mode (min{dE_1_}), but for the vHI-mode is varied between this narrowest value and the maximum QENS energy-transfer (i.e. max{dE_1_}=max{∆E}). Neutrons from the primary monochromator are scattered by the sample with which they exchange energies ∆E with a probability depending on the scattering function of the sample. In our case this is a Lorentzian function centred at zero energy-transfer and with HWHM of 0.066 meV (corresponding to a relaxation time of 10 ps). Neutrons scattered by the sample pass the secondary spectrometer with the same probability function as the primary spectrometer at its narrowest value (i.e. dE_2_ = min{dE_1_}), to arrive at a detector that has 100% efficiency.

For QENS the centre of the Gaussian function describing the primary spectrometer, E_1_, is changing on a list of n = 2048 values between −2.22 to +2.22 meV with constant energy bin-size, while the HWHM of the distribution (associated to the energy resolution) is fixed to a constant value, dE_1_ = 0.02 meV. The QENS spectrum was then obtained by centring the Gaussian of the primary spectrometer at each of these 2048 channels with equal probability. As a result, for each point ∆E of the final QENS spectrum there is an associated “configuration” of the primary spectrometer with a Gaussian centred at an energy E_1_ = E_2_ + ∆E. This is to simulate the varying incident-energy of standard backscattering QENS spectrometers. N is the number of neutrons for each “configuration” of the primary spectrometer. The E_2_ is the characteristic energy of the secondary spectrometer fixed at a constant value for all the acquisition of the QENS spectrum. For simplicity, we have encoded the energy transfer, ∆E, directly in E_1_ by setting E_2_ to zero.

To access the vHI profile, the portion of elastic scattering from the system is computed as a function of the energy-HWHM of the primary spectrometer (energy resolution), which is associated to an observation time, t_obs_^32^. As a result, in the vHI mode the HWHM of the Gaussian function describing the primary spectrometer, dE_1_, is changing on a list of n = 2048 values between 0.02 meV and 2.18 meV with constant observation time bin-size, while the centre of the distribution is fixed to a constant value on the elastic peak (E_1_ = 0) to allow the solely “elastic” events to be computed, i.e. E_2_ = E_1_. The vHI profile was then obtained by varying the Gaussian HWHM of the primary spectrometer at each of these 2048 values with equal probability. As a result, for each point *t*_*obs*_ of the final vHI profile there is an associated “configuration” of the primary spectrometer with a Gaussian HWHM of dE_1_ comprised between two limiting values. The longest observation time, corresponding to the inverse of the best energy resolution (narrowest HWHM), is max{t_obs_} = 1.66/min{dE_1_} = 55 ps, and the shortest observation time, corresponding to the inverse of the broadest energy resolution (largest HWHM), is min{t_obs_} = 1.66/max{dE_1_}=0.5 ps. The 1.66 factor for the proper conversion from HWHM to observation time has been explained in our previous work^32^ (as well as the “0.6579 meV ps” factor for the proper conversion from energy to time). Also, in the vHI mode, N is the number of neutrons for each “configuration” of the primary spectrometer.

For more detail on the *modus operandi* of neutron backscattering spectrometers, and on how get either QENS or vHI from those, please refer to the “Extra details on the methodology” section at the bottom of this letter.

## Results and Discussion

In the following sections we will present three comparisons between QENS and vHI: firstly total counts, secondly counting errors, and finally the extracted I(t) functions.

### Comparing the number of total counts

**Figure 2:**
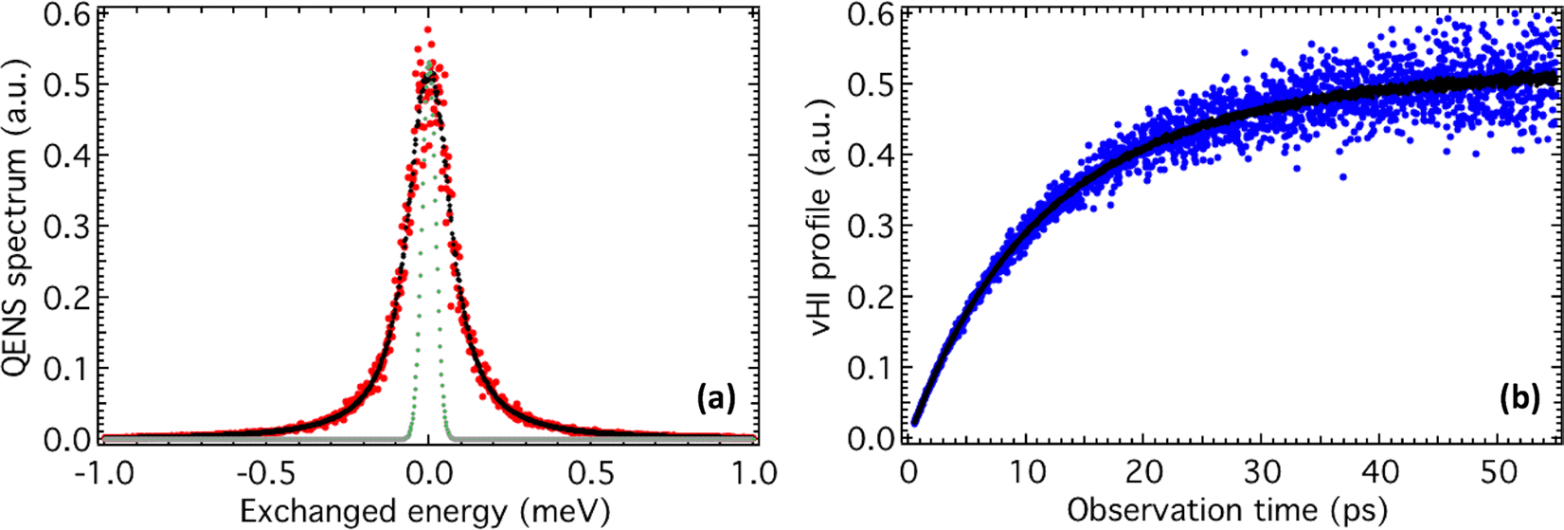
“Standard” vs “perfect” outputs. Standard QENS spectrum (panel **a**, red) and standard vHI profile (panel **b**, blue) compared with perfect QENS spectrum (panel **a**, black) and perfect vHI profile (panel **b**, black), respectively. The energy resolution used in QENS mode is also reported for clarity (panel **a**, grey). Here both QENS and vHI outputs are normalised, but in the raw data vHI scores 100 times more total counts over QENS. The number of neutron events (for each configuration of the primary spectrometer), N, was 10,000 and one million for “standard” and “perfect” outputs, respectively.

A first comparison of the two methods can be obtained by comparing the total counts at the detector for QENS and vHI modes (Fig. 2).

As a result, the total counts of the vHI method is about 100 times more than the total counts of the QENS (depending slightly on the details of the input function). The origin of this gain in total counts is because (i) the vHI method operates in the “elastic” regime which is usually at least an order of magnitude more probable than “inelastic” processes, and because (ii) for the vHI method the primary energy resolution, dE_1_, is relaxed to match the time-scale being measured (in contrast, for QENS, dE_1_ is fixed at the narrowest value). The origin of these two count-gains, the “elastic gain” and the “relaxed-resolution gain”, respectively, can be visualised by comparing Fig. 1b with Fig. 1c. By computationally removing the “relaxed-resolution count-gain” contribution, it was possible to distinguish that the “elastic count-gain” contributes a factor of about 20 to the overall count-gain.

In fact, it is also possible to further increase the count-gain of vHI by adjusting the energy-width of the secondary spectrometer, dE_2_. It turns out that the vHI approximation holds for secondary spectrometer energy-widths up to four times smaller than the energy-width of the primary spectrometer^32^. This “secondary resolution count-gain” has been computed with our MC code by adjusting the energy-width of the secondary spectrometer to be four times smaller than that of the primary. With this additional gain in place, the total count-gain factor of vHI over QENS can reach around 250. For simplicity, this “secondary resolution count-gain” will not be used in the following comparisons.

### Comparing the counting errors/statistical significance of the raw outputs

Counting advantages may not translate into any advantage in understanding the system dynamics, and indeed if the additional counts turn out to be some form of “background” they can even be a disadvantage. A straightforward way of assessing this is to compute the counting errors of both QENS and vHI outputs, which will give a measure of the statistical significance of the two methods. To do so we compare the outputs of the simulation over a given counting time, with the same output counting for “infinite” time (where “infinite” is “very long”). This can be seen as comparing with a perfect instrument without any counting error in the measured functions (Fig. 2, black dots). To estimate the counting errors, we have then computed the “relative difference” between the “standard” QENS and vHI outputs with the respective “perfect” outputs, which is related to the relative error:

1 $${\rm{relative}}\,{\rm{difference}}({\rm{x}})=\frac{{\rm{standard}}\,{\rm{output}}\,({\rm{x}})-{\rm{perfect}}\,{\rm{output}}\,({\rm{x}})}{{\rm{perfect}}\,{\rm{output}}\,({\rm{x}})}$$

**Figure 3:**
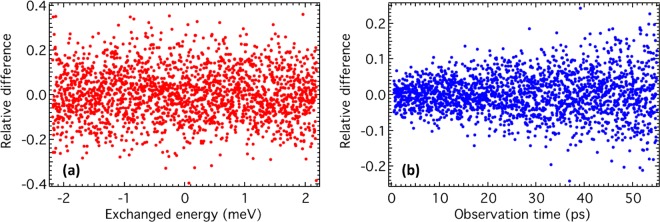
Relative difference between “standard” and “perfect” outputs for QENS (**a**) and vHI (**b**). Note the different scales.

The relative differences are a function of the energy exchange for QENS and of the observation time for vHI. Figure 3a shows the relative difference in the case of QENS, and Fig. 3b for vHI. In both cases the distribution is symmetric around zero, illustrating the absence of systematic errors. It turned out that the relative error is always below 20% in the case of vHI with an average of about 4.4%. For QENS the relative error is up to 40% and its average is about 9.2%. From the ratio between the average relative errors, we can conclude that vHI has 2 times better statistics than QENS for the same incident neutron flux.

The overall difference between standard and perfect outputs is also given by an R-Factor:

2 $${\rm{R}}-{\rm{Factor}}=\frac{\sum ||{\rm{standard}}\,{\rm{output}}\,({\rm{x}})|-|{\rm{perfect}}\,{\rm{output}}\,({\rm{x}})||}{\sum |{\rm{perfect}}\,{\rm{output}}\,({\rm{x}})|}$$

The R-Factors turned out to be 0.094 and 0.048 for QENS and vHI, respectively. The ratio of these confirms a gain in statistical relevance of 2 for vHI, which translates into QENS requiring 4 times longer counting times to achieve the same statistical relevance as vHI.

### Comparison of the extracted I(t)

These results are encouraging for vHI, particularly when we consider that results are already in the time domain. However, it is an integral approach and we need to compare how I(t) vary when computing these starting from the “standard” outputs compared with the “perfect” outputs to judge these two methods at I(t)-level.

A standard procedure to access the I(t) from the measured QENS spectrum is a numerical time-FT, then divide this by the numerical time-FT of the energy resolution function. However, since here we are interested in assessing precision rather than accuracy, we do not correct by the energy resolution, something that will anyway decrease the statistical significance of QENS-extracted I(t). The procedure to access the I(t) from the measured vHI profile is to obtain a polynomial time-derivative: basically, mapping the measured data in the power of time and then plot its derivative^32^. In this case there is no need of any energy-resolution correction.

**Figure 4:**
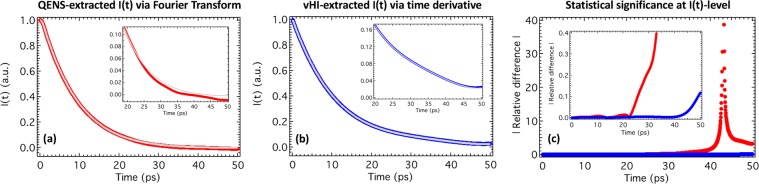
I(t) profiles extracted from “standard” QENS by numerical FT (panel **a**, red), and from “standard” vHI by polynomial time-derivative (panel **b**, blue) compared with the “perfect” I(t) profiles (panels a and b, grey). In the insets of panels (**a**) and (**b**) a zoom showing more clearly the differences between “standard” and “perfect” cases is presented. (**c**) Relative differences between “standard” and “perfect” I(t) profiles extracted from QENS by time-FT (red), and from vHI by the polynomial time-derivative method (blue). The inset of panel (**c**) is a zoom of the lower region which shows clearly the real advantage of vHI over QENS at I(t)-level.

The essential question that we address here is: which distorts the data more, FT (Fig. 4a) or polynomial derivative (Fig. 4b)? For this, we have calculated the (absolute value of the) relative differences of Eq. (1) between the (numerical) extracted I(t) from the “standard” outputs and the (numerical) extracted I(t) from the “perfect” outputs (Fig. 4c). For vHI, the relative error is basically null from zero to 45 ps, then increases almost linearly reaching its maximum of 10% at the edge of the accessible observation time window (i.e. 50 ps); its average is of 1%. For QENS, the relative error is almost negligible (i.e. below 2%) from zero to 20 ps, but it then rapidly increases to 4000% at about 45 ps, eventually relaxing to about 400% at the edge of the accessible observation time window (i.e. 50 ps); its average is of 200%. The ratio of these average relative errors gives a gain in statistical relevance of 200 for vHI.

The overall difference between standard and perfect outputs is also given by an R-Factor (Eq. 2). The R-factors turned out to be 0.018 and 0.002 for QENS and vHI, respectively. Their ratio gives a gain in statistical relevance of 10 for vHI, which would translate into QENS requiring 100 times longer counting times to achieve the same statistical relevance as vHI at I(t)-level.

We have obtained overall numbers to judge the precision. Although FT and polynomial derivatives distort the errors by smoothing the data, by comparing the ratio of the R-Factors with the ratio of average relative errors, we can conclude that the vHI method together with the polynomial time-derivative method retains at least a 10 times better statistical significance than the QENS together with the FT approach to I(t), which translates into QENS requiring at least 100 times longer counting times to achieve the same statistical relevance as vHI at I(t)-level.

## Conclusions and remarks for the future

This study provides the first evidence of the real advantage of vHI over QENS in number of counts, counting errors and discrimination at the I(t)-level.

Recently we proposed a new neutron scattering method for dynamics, i.e. the vHI method^32^. Whereas the standard neutron method for dynamics, QENS, access system dynamics by computing “inelastic” processes, our new vHI method operates entirely in the “elastic” regime and accesses system dynamics by relaxing the energy resolution of one of the two resolution components of the spectrometer. In our former work^32^ we numerically calculated the vHI profile on a set of three representative input functions (i.e. single exponential, double exponential and stretched exponential), and we show that in all cases our vHI method reproduces the input functions as well as the common QENS accurately. In this letter we investigate the “precision” of these two methods for dynamics. Essentially, how much useful information do we get from each method for the same incident neutron flux. To do so, we developed an *ad hoc* MC code that has the option to operate the primary spectrometer either in QENS mode or in vHI mode. As a result, both QENS spectra and vHI profiles have been generated under the same “experimental” conditions, i.e. same number of incoming neutrons, same instrument layout, same sample input function; the only difference being in the “use” of the primary spectrometer.

The first comparison was on the total number of counts, i.e. the total number of neutrons collected at the detector bank on the same set-up in the two modes. In the case of vHI there were about 100 times more neutron counts. The second aspect was to see if and how these extra counts transformed into better raw information. The counting errors on the measured outputs, QENS spectrum and vHI profile respectively, have been computed. It turned out that the vHI profile has 2 times better statistics than the QENS spectrum, which translates into QENS requiring 4 times longer counting times to achieve the same statistical relevance as vHI. Finally, the comparison on the extracted I(t) has been done as well. The relative error on the numerical time-FT of the QENS spectrum has been compared with that of the polynomial time-derivative of the vHI profile. It resulted that the I(t) extracted from the vHI profile has at least 10 times better statistics than the I(t) extracted from the QENS spectrum, which translates into QENS requiring 100 times longer counting times to achieve the same relevance as vHI at I(t)-level.

We wish to stress the following:

- First that these “vHI advantage over QENS” has been obtained having operate QENS at its ideal condition, i.e. the instrumental energy uncertainty (the energy resolution of the two spectrometers) has been chosen to be small enough to allow our MC *in silico* QENS spectrometer to resolve the dynamics of the system entirely. In this sense, this is the most probable and relevant condition in practice.
- The focus of this letter is to judge the relative significance of standard experimental data from QENS and vHI to their ideal outputs. As a result, the comparisons have been done against the output of “perfect” spectrometers.
- Perhaps the best comparison made in this letter is the “raw” outputs of these two methods, i.e. QENS spectrum and vHI profile. Considering that both can be fitted with physical models to extract information on the dynamics of the systems under investigations, we believe that the advantages of vHI over QENS are genuine.
- On the other hand, the comparison presented in this letter of the extracted I(t) required the use of either a time-FT for the QENS or a polynomial time-derivative for the vHI. These two procedures smooth the data, preventing direct comparison of their counting statistics. However, the advantages of vHI over QENS are also clear for the extracted I(t) case.
- We note, finally, that with sufficiently high counting statistics a simple numerical derivative of the measured vHI can be used to obtain a very precise I(t), but this option never arises for QENS even at very high counting statistics.

## Extra details on the methodology

In this section we present an overview of the general layout of any neutron scattering (backscattering) experiments, as routinely used for QENS, and how this can be used in vHI mode. In doing so, a comparison between the two methods for measuring dynamics is presented. For more details on vHI please refer to our previous works^22,32^, for QENS to Ref. ^12–16^ and the huge literature on the subject.

### General layout of a QENS backscattering spectrometer

The layout of standard backscattering QENS spectrometers is reported in Fig. 1a. We chose this set-up for QENS for simplicity and because, consistently with our previous works^22,32^, it can be adapted to work in vHI mode. Neutrons coming from a source (reactor or spallation based) reach the primary spectrometer that selects a monochromatic beam with an energy distribution that is known as primary energy resolution. In real cases this is often a Gaussian function with a width related to the energy uncertainty of the neutrons. These neutrons reach the sample with which they exchange energy and momentum. These scattered neutrons reach the secondary spectrometer which acts as a filter, typically identical to the energy distribution of the primary monochromator. The filter only reflects neutrons within that energy distribution into the detector.

### Elastic windows scan

If the characteristic energies of primary and secondary crystal spectrometers are the same, only neutrons that do not exchange energy with the sample (within the energy resolution of the instrument), i.e. the “elastically scattered” neutrons, will reach the detector bank. This operational mode is available on any QENS backscattering spectrometers and is often used before a proper QENS experiment to gain preliminary information, and it is known as “elastic fixed window scan”.

### The QENS method

Alternatively, if the characteristic energies of primary and secondary crystal spectrometers differ by ∆E, then only neutrons that exchange energy with the sample corresponding exactly to this energy difference ∆E (within the energy resolution of the instrument) will reach the detectors. These are inelastically scattered neutrons. The QENS method is based on the measurement of these inelastically scattered neutrons as a function ∆E, i.e. the energy difference between the characteristic energies of the two crystal spectrometers. Figure 1b show how a QENS spectrum is composed of inelastic measurements. Inelastic neutron scattering was proposed by Brockhouse^13^ for which he received the Nobel Prize in Physics in 1994, and it is used since then. As a result, the output of a QENS experiment (i.e. a QENS spectrum) reports the number of scattered neutrons as a function of their energy transfer with the sample (e.g. Fig. 2a). In this letter, we have neglected the spatial dependence of the spectrum, which adds nothing to our discussion.

To extract the information on the dynamics of the system, a QENS model function is chosen to fit the measured QENS spectrum. Usually, a single Lorentzian function or linear combination of two or more Lorentzian functions are used, in which the widths are inversely proportional to the relaxation times of the dynamical processes in the system. An alternative route is to time-FT the measured QENS spectrum, to obtain the associated van Hove intermediate scattering function, I(t). A model function is then used to fit the I(t) to access the information on the systems dynamics. As part of the fitting procedure the measured QENS spectrum is usually deconvoluted to take account of the finite energy resolution of the spectrometers. Alternatively, I(t) can be obtained by dividing the FT of the measured QENS by the FT of the energy resolution QENS spectrum of the spectrometer. The energy resolution QENS spectrum of the spectrometer can be obtained by measuring the QENS spectrum of a vanadium sample, which is a near-perfect incoherent elastic scatterer and/or by measuring the QENS spectrum of the sample at very low temperature, e.g. below 10 K, where no dynamical processes arise.

### Our vHI method

Recently, we introduced a new neutron scattering method^32^ to access system dynamics, which we denoted the “vHI method”, which stands for “van Hove integral”. Basically, the measured output (e.g. Fig. 2b) is the running-time integral of the van Hove intermediate scattering function, I(t). This gives a direct way to access I(t) in the time domain without Fourier transforms, *a priori* modelling, or energy-resolution corrections. The vHI profile can, in turn, be fitted with model functions directly or mapped in the vector space of the powers of time by a polynomial interpolation to give access to the I(t).

This vHI method can be based on the set-up of backscattering machines as reported in Ref. ^22,32^. Contrary to QENS, it operates at the elastic condition, i.e. at the fixed energy exchange value ∆E = 0, and so without scanning any energy exchange. This means that in vHI the characteristic energy of the primary crystal spectrometer matches that of the secondary spectrometer. In vHI the dynamics is probed instead by scanning the energy band-width of one of the two spectrometers, having the other at a fixed energy resolution value. Figure 1c show how a vHI profile is composed of elastic measurements.
